# A Mixed Approach to Similarity Metric Selection in Affinity Propagation-Based WiFi Fingerprinting Indoor Positioning

**DOI:** 10.3390/s151127692

**Published:** 2015-10-30

**Authors:** Giuseppe Caso, Luca de Nardis, Maria-Gabriella di Benedetto

**Affiliations:** Department of Information Engineering, Electronics and Telecommunications (DIET), Sapienza University of Rome, Via Eudossiana 18, 00184, Rome, Italy; E-Mails: lucadn@newyork.ing.uniroma1.it (L.N.); gaby@newyork.ing.uniroma1.it (M.-G.B.)

**Keywords:** WiFi fingerprinting indoor positioning, similarity metrics, W*k*NN, affinity propagation

## Abstract

The weighted *k*-nearest neighbors (W*k*NN) algorithm is by far the most popular choice in the design of fingerprinting indoor positioning systems based on WiFi received signal strength (RSS). W*k*NN estimates the position of a target device by selecting *k* reference points (RPs) based on the similarity of their fingerprints with the measured RSS values. The position of the target device is then obtained as a weighted sum of the positions of the *k* RPs. Two-step W*k*NN positioning algorithms were recently proposed, in which RPs are divided into clusters using the affinity propagation clustering algorithm, and one representative for each cluster is selected. Only cluster representatives are then considered during the position estimation, leading to a significant computational complexity reduction compared to traditional, flat W*k*NN. Flat and two-step W*k*NN share the issue of properly selecting the similarity metric so as to guarantee good positioning accuracy: in two-step W*k*NN, in particular, the metric impacts three different steps in the position estimation, that is cluster formation, cluster selection and RP selection and weighting. So far, however, the only similarity metric considered in the literature was the one proposed in the original formulation of the affinity propagation algorithm. This paper fills this gap by comparing different metrics and, based on this comparison, proposes a novel mixed approach in which different metrics are adopted in the different steps of the position estimation procedure. The analysis is supported by an extensive experimental campaign carried out in a multi-floor 3D indoor positioning testbed. The impact of similarity metrics and their combinations on the structure and size of the resulting clusters, 3D positioning accuracy and computational complexity are investigated. Results show that the adoption of metrics different from the one proposed in the original affinity propagation algorithm and, in particular, the combination of different metrics can significantly improve the positioning accuracy while preserving the efficiency in computational complexity typical of two-step algorithms.

## 1. Introduction

Indoor positioning is nowadays an important and interesting research topic, as it promises to enable the extension of outdoor location-based services to indoor environments [1]. Among many proposed and investigated approaches and systems [2], the fingerprinting technique, based on the collection of received signal strength (RSS) values from WiFi access points (APs) detected in the area of interest, is one of the most popular ones [3]. Fingerprinting operates in two phases: offline and online. During the offline phase, RSS values (fingerprints) are collected at previously-selected positions, referred to as reference points (RPs). During the online phase, the location of target devices in unknown positions is estimated as a function of the positions of the RPs that best match the RSS values measured by the devices (online readings), according to a specified similarity metric.

Among fingerprinting-based solutions, weighted *k*-nearest-neighbors (W*k*NN) algorithms, in which the estimated position is obtained as the weighted average of *k* selected RPs positions, are by far the most widely investigated. W*k*NN algorithms are usually divided into deterministic and probabilistic ones: the first ones are highly appealing, because, thanks to the use of deterministic similarity metrics computable by comparing the online reading and each RP fingerprint, they are relatively easy to implement [4,5,6,7]; on the other hand, W*k*NN probabilistic algorithms can improve the performance of deterministic ones, at the price of higher implementation complexity [2,3], since they are based on the estimation of the signal strength distribution for each (AP, RP) pair and on the computation of likelihood probabilities, derivable through the comparison between the online reading and the estimated RSS distributions [8,9,10,11].

The accuracy and complexity of fingerprinting algorithms depend on two main issues: (1) careful planning of the offline phase; and (2) the definition and implementation of algorithms adopted in the online phase. Focusing in particular on the online phase, which is also the main subject of this paper, the accuracy can be improved by implementing an optimal RP selection through a proper definition of both the similarity metric and the value of *k*; at the same time, system complexity can be decreased by reducing the number of online operations requested for obtaining a position estimation. Within this context, previous works proposed the adoption of two-step algorithms, which foresee a preliminary RP clustering step during which the RPs are divided into clusters. Clusters are then taken into account in the online phase by organizing it into two-step, referred to as coarse and fine localization [6,9,12]. During the coarse localization step, the online reading is compared to the fingerprints of each RP cluster according to the selected similarity metric; only the clusters passing a predefined similarity threshold are then selected. The main goal of the coarse localization step is to reduce the computational complexity of the following fine localization step, by reducing the RP space. In fact, while for a generic W*k*NN flat algorithm, all of the RPs are compared to the online reading in order to select the *k* most relevant RPs, in two-step algorithms, only the RPs belonging to the selected clusters are taken into account.

Several previous works proposed the use of the affinity propagation algorithm [13] for the creation of the clusters. The affinity propagation algorithm exploits the evaluation of mutual similarities between the RPs [12,14,15,16]. As a result, in two-step algorithms adopting the affinity propagation clustering, the impact of the similarity definition is two-fold, both in the RP clustering and cluster selection steps.

Moving from the previous observations, this work analyzes the performance of flat and two-step affinity propagation-based algorithms, focusing on W*k*NN deterministic solutions.

The positioning accuracy of two-step algorithms is evaluated by analyzing the impact of different similarity metrics at all steps of the position estimation procedure: RP clustering in the offline phase and coarse and fine localization steps in the online phase. The impact of the similarity metrics on flat algorithms is investigated, as well, in order to provide a benchmark for two-step algorithms. Moreover, flat and two-step algorithms are compared in terms of computational complexity, by evaluating the average number of online operations required to obtain a position estimate.

The choice of restricting the analysis to deterministic metrics is due to two main reasons: (1) to guarantee a clear analysis of the impact of the combination of different metrics by focusing on a homogeneous family of metrics; and (2) to verify the assumption that two-step algorithms can reduce computational complexity even for metrics that in origin already guarantee a low complexity. Extension of the analysis to probabilistic metrics will be addressed in future works.

Performance evaluation is carried out by means of experimental results obtained in the testbed implemented at the Department of Information Engineering, Electronics and Telecommunications (DIET) of Sapienza University of Rome.

The rest of the paper is organized as follows. Section 2 introduces a general model for W*k*NN fingerprinting-based indoor positioning systems, valid for both flat and two-step algorithms, and discusses next the role of *k* by introducing fixed and dynamic selection schemes. Then, Section 2 focuses on two-step algorithms using the affinity propagation clustering algorithm, by reviewing the algorithm and discussing its application to the RP clustering and coarse localization steps. Section 3 analyzes in detail the role of the similarity metric in fingerprinting-based algorithms: first, Section 3.1 and Section 3.2 introduce the similarity metrics considered in this work, distinguishing between metrics suitable for both offline and online phases and metrics only usable in the offline case. Section 3.3 completes the analysis by introducing a comparative framework for the online similarity metrics. Next, Section 4 describes the testbed implemented at DIET and presents experimental results focusing on the comparative analysis of the metrics and algorithms, in terms of topology, positioning accuracy and computational complexity. Finally, Section 5 concludes the paper and discusses future research lines.

## 2. System Model

### 2.1. A General Model for WkNN Deterministic Algorithms

Both flat and two-step deterministic W*k*NN algorithms, introduced in Section 1, can be described by the following general model.

Given an area of interest, denoting with *L* the total number of WiFi APs (${\mathrm{AP}}_{l}$ with l=1,2,⋯,L) that can be detected and with *N* the number of RPs (${\mathrm{RP}}_{n}$ with n=1,2,⋯,N), during the fingerprinting offline phase *N*, L×1 vectors are defined, with the generic vector $s_{n}$ containing in its $s_{l,n}$ component the RSS measured for the (l,n) AP-RP pair.

In the general case of two-step algorithms, an offline clustering step takes place after the RSSs’ collection, during which the RPs are divided into $N_{c}<N$ clusters and a particular RP is elected as the clusterhead for each cluster.

Once the offline phase is complete, the position estimation is obtained during the online phase, divided into coarse localization (cluster selection/matching) and fine localization (RP selection and weighting) steps. Denoting with $s_{i}$ the RSS vector measured for the *i*-th online reading, the coarse localization step selects the $N_{c,i}\leq N_{c}$ clusters that best match the online reading; this is obtained through the computation of $N_{c}$ similarity values ${\mathrm{sim}}_{i,n_{c}}^{C}={\mathrm{sim}}^{C}\left(s_{i},s_{n_{c}}\right)$ (with $n_{c}=1,2,\cdots ,N_{c}$) between the online reading and a fingerprint selected as the $n_{c}$-th cluster’s representative, where ${\mathrm{sim}}_{i,n_{c}}$ is a properly-defined similarity metric and superscript *C* indicates that such similarity values are evaluated during the coarse localization step. Denoting with $N_{i}$ the total number of RPs belonging to the $N_{c,i}$ selected clusters, during the fine localization step, the first *k* out of $N_{i}$ RPs are selected by computing $N_{i}$ similarity values ${\mathrm{sim}}_{i,n}^{F}={\mathrm{sim}}^{F}\left(s_{i},s_{n_{i}}\right)$ (with $n_{i}=1,2,\cdots ,N_{i}$) between the online reading and the RP fingerprints, where ${\mathrm{sim}}_{i,n}$ is a properly-defined similarity metric and superscript *F* indicates that such similarity values are evaluated during the fine localization step. Next, the *k* selected RPs are used in the W*k*NN-based position estimation formula:

(1) $${\hat{p}}_{i}=\frac{\sum_{n=1}^{k}\left({\mathrm{sim}}_{i,n}^{F}\right)p_{n}}{\sum_{n=1}^{k}{\mathrm{sim}}_{i,n}^{F}}$$

where $p_{n}=\left(x_{n},y_{n},z_{n}\right)$ and ${\hat{p}}_{i}=\left({\hat{x}}_{i},{\hat{y}}_{i},{\hat{z}}_{i}\right)$ are the position of the *n*-th RP and the estimate of the unknown *i*-th position in the 3D coordinates system defined for the area of interest, respectively.

Flat algorithms are included in the above model by selecting $N_{c}=N_{i}=N$, that is assuming a clustering algorithm that leads to *N* one-dimensional clusters and a coarse localization step that selects all clusters. The *k* RPs to be considered in Equation (1) are then selected through the evaluation of *N* similarity values ${\mathrm{sim}}_{i,n}^{F}={\mathrm{sim}}^{F}\left(s_{i},s_{n}\right)$ (with n=1,2,⋯,N).

A few open points remain in the complete definition of the model, which are introduced in the following and will be analyzed in detail later on in the paper:

- Selection of *k*: Previous works showed the impact of the methodology of selecting the set of *k* RPs and the value of *k* itself on the positioning performance of deterministic W*k*NN algorithms [5,6,7], but no univocal and general way to select the value of *k* was provided. Section 2.2 presents the two selection schemes considered in this work.
- Clustering algorithm: Two-step algorithms considered in this work require the selection of a clustering algorithm to be used in the offline phase. Several works proposed the affinity propagation algorithm [13] as a viable solution to fulfill this task [12,14,15,16], and the algorithm was also adopted in this work. The affinity propagation algorithm and its application to RP clustering and coarse localization steps are discussed in Section 2.3.
- Similarity metrics: Different similarity metrics can be adopted in two-step algorithms for coarse and fine localization steps, respectively, leading to ${\mathrm{sim}}_{i,n}^{C}\neq {\mathrm{sim}}_{i,n}^{F}$, and thus, introducing an additional degree of freedom in the algorithm design. This possibility, not yet explored in the literature, will be thoroughly addressed in Section 3.

### 2.2. k Selection Schemes

In [4,6,7], among many others, an *a priori* fixed *k* scheme was proposed, in which *k* is selected as a system parameter and does not change during the online estimation requests. It can be easily shown that the choice of a fixed *k* value is not optimal, since the value of *k* that minimizes the estimation error will in general depend on the target position. Even finding a fixed value of *k* that minimizes the average error on all possible target positions is not trivial. Experimental studies found, however, that a value of *k* between 2 and 10 leads to an average error close to its minimum achievable value [3,5,6,7,17,18].

This empirical result seems reasonable by considering that, on the one hand, if k=1 is selected, each positioning request is solved by assigning to the target device the position of the best matching RP, and this could lead to high positioning errors for some target positions. On the other hand, as *k* increases, more and more RPs are included in Equation (1), eventually including some with low similarity and, thus, decreasing the positioning accuracy. The impact of a fixed *k* selection scheme on the positioning accuracy of flat algorithms is investigated in Section 4.2.

In order to overcome the issues inherent to the fixed *k* scheme, several works proposed a dynamic *k* scheme, in which the value is adjusted at each positioning request [5,18,19]. In general, this scheme relies on the definition of a threshold *λ* taking values in the same domain of the similarity metric and on the selection of the RPs that show a value of the metric above the threshold. Additional flexibility can be achieved by introducing a variable threshold. In this case, given the *i*-th positioning request, *λ* is evaluated as a function of the RPs similarity values ${\mathrm{sim}}_{i,n}^{F}$, that is:

(2) $$\lambda =\lambda_{i}\left({\mathrm{sim}}_{i,n}^{F}\right)=f\left({\mathrm{sim}}_{i,n}^{F}\right)$$

where f(.) is a function of the observed similarity values and $n=1,2,\cdots ,N_{i}$.

This scheme is investigated in conjunction with two-step algorithms in Section 4, where the reader can also find additional details on the implementation of Equation (2).

### 2.3. Affinity Propagation Clustering for Indoor Positioning

#### 2.3.1. RP Clustering

The affinity propagation algorithm is a clustering algorithm that divides a set of elements into clusters and elects for each cluster a representative clusterhead, also dubbed the exemplar [13]. Differently from traditional *k*-center clustering algorithms that usually start the iterative procedure for clusters creation and exemplar election by choosing both the number of output clusters and a corresponding random set of initial exemplars, affinity propagation starts by granting each element the same chances to become an exemplar, thus removing the dependence from the initial conditions.

The algorithm follows a distributed and iterative approach: elements are seen as network nodes, which exchange messages containing computed values that measure the affinity that one element has for choosing another element as its exemplar, until it converges to a stable set of exemplars and corresponding clusters.

In the context of indoor positioning, since elements correspond to RPs, the algorithm can be implemented in a centralized way where no actual message transmission is required, and for each iteration, the requested values are evaluated by a central processing unit based on an initial measure of similarity ${\mathrm{sim}}^{CL}\left(s_{n_{1}},s_{n_{2}}\right)$ (with $n_{1},n_{2}=1,2,\cdots ,N$ and $n_{1}\neq n_{2}$) between each RP pair, where the superscript CL indicates that such similarity values are evaluated during the clustering step, measuring how well the ${\mathrm{RP}}_{n_{2}}$ is suited to be the exemplar for ${\mathrm{RP}}_{n_{1}}$.

The self-similarity value ${\mathrm{sim}}^{CL}\left(s_{n},s_{n}\right)$ (with n=1,2,⋯,N), that is also dubbed the preference, indicates the possibility that ${\mathrm{RP}}_{n}$ may become an exemplar. In order to give all RPs the same chance to become an exemplar, their preferences are initially set to a common finite value, typically defined as:

(3) $$\mathrm{pref}\left(s_{n}\right)={\mathrm{sim}}^{CL}\left(s_{n},s_{n}\right)=\gamma \cdot \mathrm{median}\left\{{\mathrm{sim}}^{CL}\left(s_{n_{1}},s_{n_{2}}\right)\right\},\;\forall n_{1},n_{2}\in \left\{1,2,\cdots ,N\right\},\;n_{1}\neq n_{2}$$

where *γ* is a tunable parameter [12,13].

The definition of exemplars relies on the evaluation of two values, ideally exchanged between RPs pairs and defined as follows:

- Responsibility $\mathrm{resp}(s_{n_{1}},s_{n_{2}})$: This reflects the accumulated evidence for how well-suited ${\mathrm{RP}}_{n_{2}}$ is to serve as the exemplar for ${\mathrm{RP}}_{n_{1}}$, taking into account other potential exemplars for ${\mathrm{RP}}_{n_{1}}$.
- Availability $\mathrm{avail}(s_{n_{1}},s_{n_{2}})$: This reflects the accumulated evidence for how appropriate it would be for ${\mathrm{RP}}_{n_{1}}$ to choose ${\mathrm{RP}}_{n_{2}}$ as its exemplar, taking into account the support from other RPs that ${\mathrm{RP}}_{n_{2}}$ should be an exemplar.

These values are iteratively updated according to the following equations:

(4) $$\mathrm{resp}\left(s_{n_{1}},s_{n_{2}}\right)={\mathrm{sim}}^{CL}\left(s_{n_{1}},s_{n_{2}}\right)-\max_{n_{3}}\left\{\mathrm{avail}\left(s_{n_{1}},s_{n_{3}}\right)+{\mathrm{sim}}^{CL}\left(s_{n_{1}},s_{n_{3}}\right)\right\}$$

(5) $$\mathrm{avail}\left(s_{n_{1}},s_{n_{2}}\right)=\min\left\{0,\mathrm{resp}\left(s_{n_{2}},s_{n_{2}}\right)+\sum_{n_{3}}\max\left\{0,\mathrm{resp}\left(s_{n_{3}},s_{n_{2}}\right)\right\}\right\}$$

$\forall n_{1},n_{2},n_{3}\in \left\{1,2,\cdots ,N\right\}$ and $n_{1}\neq n_{2}$, $n_{3}\neq n_{2}$ in Equation (4) and $n_{3}\neq n_{1},n_{2}$ in Equation (5).

In order to facilitate the convergence of the iterative procedure and to avoid ringing oscillations, a damping factor (DF) ∈[0.5,1) is typically introduced, leading to the following expressions for the new values of responsibility and availability:

(6) $$\begin{array}{cc} {\mathrm{resp}}_{new}\left(s_{n_{1}},s_{n_{2}}\right) & =DF\cdot {\mathrm{resp}}_{old}\left(s_{n_{1}},s_{n_{2}}\right)+\left(1-DF\right)\cdot {\mathrm{resp}}_{new}^{\prime }\left(s_{n_{1}},s_{n_{2}}\right)\\ {\mathrm{avail}}_{new}\left(s_{n_{1}},s_{n_{2}}\right) & =DF\cdot {\mathrm{avail}}_{old}\left(s_{n_{1}},s_{n_{2}}\right)+\left(1-DF\right)\cdot {\mathrm{avail}}_{new}^{\prime }\left(s_{n_{1}},s_{n_{2}}\right) \end{array}$$

$\forall n_{1},n_{2}\in \left\{1,2,\cdots ,N\right\}$, $n_{1}\neq n_{2}$, with ${\mathrm{resp}}_{new}^{\prime }\left(s_{n_{1}},s_{n_{2}}\right)$ and ${\mathrm{avail}}_{new}^{\prime }\left(s_{n_{1}},s_{n_{2}}\right)$ evaluated by using Equations (4) and (5), respectively.

Two main issues were identified in the application of the affinity propagation algorithm in [12,13]:

- Degeneracies: Degeneracies can arise, for example, if the similarity metric is commutative and two elements (RPs) are isolated from all of the others. In this case, oscillations in deciding which of the two elements should be the exemplar might appear. The solution proposed in [13] is to add a small amount of random noise to similarities values to avoid such a deadlock situation.
- Outliers: The algorithm might occasionally lead to an RP belonging to a cluster, but being physically far away from the cluster exemplar. In [12], taking advantage of the knowledge of the position of each RP, each outlier is forced to join the cluster characterized by the exemplar at the minimum distance from the outlier itself.

#### 2.3.2. Cluster Selection

The main goal of the coarse localization or cluster selection step is to reduce the RP space, through the selection of a subset of clusters that match the online reading, according to a specified similarity metric. This reduces the computational complexity of the subsequent fine localization step and, furthermore, may lead to an improvement in positioning accuracy by discarding possible outliers [12].

In this paper, the criteria proposed in [12] for the coarse localization step are adopted. Such criteria are defined as follows:

- Similarity to the exemplar fingerprints (Criterion I): the similarity ${\mathrm{sim}}_{i,n_{c}}^{C}$ between the *i*-th online reading and each $n_{c}$-th exemplar fingerprint (with $n_{c}=1,2,\cdots ,N_{c}$) is evaluated, and clusters corresponding to exemplars with similarity values above a predefined threshold *α* are selected.
- Similarity to the cluster average fingerprints (Criterion II): in this case, a cluster fingerprint is computed by averaging the RP fingerprints within the cluster. The similarity ${\mathrm{sim}}_{i,n_{c}}^{C}$ between the *i*-th online reading and each $n_{c}$-th cluster fingerprint (with $n_{c}=1,2,\cdots ,N_{c}$) is then evaluated, and the clusters with similarity values above *α* are selected.

The value of *α* should be selected so as to avoid either the selection of too few or too many clusters that might lead to high positioning errors if wrong clusters are selected, on the one hand, and negligible reduction of the RP space, on the other. In this paper, the following definition of *α*, provided in [12], is adopted:

(7) $$\alpha =\alpha_{1}\cdot \max_{e\in E}\left\{{\mathrm{sim}}^{C}\left(s_{i},e\right)\right\}+\alpha_{2}\cdot \min_{e\in E}\left\{{\mathrm{sim}}^{C}\left(s_{i},e\right)\right\}$$

where *E* contains: (1) the set of exemplar fingerprints when Criterion I is adopted; or (2) the set of cluster fingerprints when Criterion II is adopted, respectively; and $\alpha_{1}+\alpha_{2}=1$. The values of $\alpha_{1}$ and $\alpha_{2}$ allow one to adjust the number of selected clusters: as an example, the smaller the number of desired selected clusters, the higher should be the value of $\alpha_{1}$ (and conversely, the lower the value of $\alpha_{2}$). The impact of adopting Criterion I *vs.* Criterion II during the coarse localization on the positioning accuracy will be analyzed in detail in Section 4.3.

#### 2.3.3. Similarity Metric for RP Clustering and Cluster Selection Steps

All previous works applying affinity propagation to indoor positioning share the same definition of pairwise similarity ${\mathrm{sim}}^{CL}\left(s_{n_{1}},s_{n_{2}}\right)$ [12,14,15,16]. This definition, inherited from [13], is as follows:

(8) $${\mathrm{sim}}^{CL}\left(s_{n_{1}},s_{n_{2}}\right)=-{\left[D^{2}\left(s_{n_{1}},s_{n_{2}}\right)\right]}^{2}\;\forall n_{1},n_{2}\in \left\{1,2,\cdots ,N\right\},\;n_{1}\neq n_{2},$$

where $D^{2}\left(s_{n_{1}},s_{n_{2}}\right)$ expresses the Euclidean distance between the RP fingerprints:

(9) $$D^{2}\left(s_{n_{1}},s_{n_{2}}\right)={\left(\sum_{l=1}^{L}{\left|s_{l,n_{1}}-s_{l,n_{2}}\right|}^{2}\right)}^{\;\;\frac{1}{2}}$$

However, the metric in Equation (8) was not proven to be optimal in the sense of both RP clustering quality and system positioning accuracy. Furthermore, to the authors best knowledge, all existing works adopt the above definition for both clustering and coarse localization steps, leading to the same metric definition for ${\mathrm{sim}}^{CL}\left(.,.\right)$ and ${\mathrm{sim}}^{C}\left(.,.\right)$. In this work, on the contrary, different definitions of similarity metrics are investigated, and combinations of the different metrics for clustering and coarse localization steps are proposed. Possible candidates for the role of similarity metrics are discussed in Section 3, and the results of the above analysis are presented in Section 4.3.

## 3. Similarity in the Context of WiFi Fingerprinting Indoor Positioning

This section reviews and defines suitable candidates for the role of similarity metrics in both flat and two-step algorithms, differentiating between metrics that are only applicable during the offline phase as ${\mathrm{sim}}^{CL}\left(.,.\right)$ and metrics that, oppositely, are applicable to both offline and online phases, as ${\mathrm{sim}}^{C/F}\left(.,.\right)$. The interested reader can refer to [17] for a comprehensive analysis of similarity metrics only focused, however, on a flat *k*NN algorithm with no weighting. For the analysis carried out in this work, a subset of such metrics was selected so as to ensure the inclusion of metrics with different characteristics in terms of expected impact on the different steps involved in a two-step algorithm (e.g., linearity *vs.* lack of it). The extension to additional metrics defined in [17] and elsewhere will be addressed in future works.

The section concludes with the definition of a comparative framework for the selected similarity metrics, used to identify the most suitable candidates for the role of the similarity metric in the experimental analysis later presented in Section 4.

### 3.1. Offline Phase Similarity Metrics

Metrics to be adopted during the offline phase can take advantage of two kinds of information regarding the RPs:

- RP positions $p_{n}$ (with n=1,2,⋯,N).
- RP RSS fingerprints $s_{n}$ (with n=1,2,⋯,N).

As a result, during the offline phase, both spatial distance-based and RSS-based similarity metrics, denoted with ${\mathrm{sim}}^{CL}\left(p_{n_{1}},p_{n_{2}}\right)$ and ${\mathrm{sim}}^{CL}\left(s_{n_{1}},s_{n_{2}}\right)$ ($\forall n_{1},n_{2}\in \left\{1,2,\cdots ,N\right\}$ and $n_{1}\neq n_{2}$), respectively, can be defined. This constitutes the main difference between the offline and online phases, since in the latter one, only RSS-based similarity metrics can be adopted as ${\mathrm{sim}}^{C/F}\left(s_{i},s_{n}\right)$. In Subsection 3.1.1, a spatial distance-based metric is proposed leaving the analysis of RSS-based similarity metrics to Section 3.2, as they are common to both the offline phase (between vectors $s_{n_{1}}$ and $s_{n_{2}}$) and the online phase (between vectors $s_{i}$ and $s_{n}$).

#### 3.1.1. A Spatial Distance-Based Similarity Metric

Moving from the assumption that the RPs in close physical proximity should exhibit a high similarity and taking advantage of the fact that the RP positions are known, a spatial distance-based similarity metric can be defined as follows:

(10) $${\mathrm{sim}}^{CL}\left(p_{n_{1}},p_{n_{2}}\right)={\left[d\left(p_{n_{1}},p_{n_{2}}\right)\right]}^{-1}$$

where $d(p_{n_{1}},p_{n_{2}})$ indicates the Euclidean distance between the coordinates vectors $p_{n_{1}}$ and $p_{n_{2}}$.

### 3.2. Offline/Online Phase Similarity Metrics

In this section, several RSS-based similarity metrics are presented as possible ${\mathrm{sim}}^{C/F}\left(s_{i},s_{n}\right)$ metrics, together with their application to the estimation formula reported in Equation (1). However, as already discussed in Section 3.1, they are applicable to RP clustering, as well as ${\mathrm{sim}}^{CL}\left(s_{n_{1}},s_{n_{2}}\right)$ metrics.

#### 3.2.1. Minkowski Distance-Based Metrics: Manhattan and Euclidean

A popular choice for defining a deterministic similarity metric is the evaluation of the Minkowski distance, with order *p*, between the online vector $s_{i}$ and each RP fingerprint $s_{n}$. Denoted as $D^{p}\left(s_{i},s_{n}\right)$, it is defined as follows:

(11) $$D^{p}\left(s_{i},s_{n}\right)={\left(\sum_{l=1}^{L}{\left|s_{l,i}-s_{l,n}\right|}^{p}\right)}^{\;\;\frac{1}{p}}\;p\geq 1$$

Orders typically used are p=1, corresponding to the Manhattan distance $(D^{1}\left(s_{i},s_{n}\right)$), and p=2, corresponding to the Euclidean distance $(D^{2}\left(s_{i},s_{n}\right)$). Note that Minkowski distance is indeed a dissimilarity metric, and its reciprocal can be adopted as a similarity definition, as proposed, for example, in [5,6,7]. Introducing the shortened notation $D_{i,n}^{p}=D^{p}\left(s_{i},s_{n}\right)$ and selecting the *k* RPs with the lower distances, the estimated position is then obtained as follows:

(12) $${\hat{p}}_{i}=\frac{\sum_{n=1}^{k}{\left(D_{i,n}^{p}\right)}^{\;-1}p_{n}}{\sum_{n=1}^{k}{\left(D_{i,n}^{p}\right)}^{\;-1}}$$

In Equation (12), the set of *k* RPs is weighted so that the RP at the minimum distance is the one with the highest impact on the position estimation.

#### 3.2.2. Inner Product-Based Metrics: Cosine Similarity and Pearson Correlation

Metrics based on modified versions of the inner product between RSS vectors are quite popular, as well. The inner product is defined as follows:

(13) $$\langle s_{i},s_{n}\rangle =\sum_{l=1}^{L}s_{l,i}s_{l,n}$$

Moving from this definition, cosine similarity (in the following referred to as $CS(s_{i},s_{n})$) was proposed in [20] and is defined as follows:

(14) $$CS\left(s_{i},s_{n}\right)=\frac{\langle s_{i},s_{n}\rangle }{\left|\right|s_{i}\left||\;|\right|s_{n}\left|\right|}=\frac{\sum_{l=1}^{L}s_{l,i}s_{l,n}}{\sqrt{\sum_{l=1}^{L}s_{l,i}^{2}}\sqrt{\sum_{l=1}^{L}s_{l,n}^{2}}}$$

where ||·|| indicates the vector $\ell_{2}$-norm. The name stems from the fact that cosine similarity can be seen as the cosine of the angle between the two vectors. Note that, although in general, CS takes values in [-1,+1], if both vectors have completely positive or negative values, it is limited between zero and one. This metric finds large application in research areas related to information retrieval, text mining and clustering schemes, and it has been proposed in the context of indoor positioning in [20,21,22,23]. According to this approach, once the $N_{i}$ (or *N*) cosine similarities are evaluated, they are used for selecting the *k* RPs most relevant to the position estimation. This leads to the following position estimation formula:

(15) $${\hat{p}}_{i}=\frac{\sum_{n=1}^{k}\left({CS}_{i,n}\right)p_{n}}{\sum_{n=1}^{k}{CS}_{i,n}}$$

where ${CS}_{i,n}=CS\left(s_{i},s_{n}\right)$.

When evaluated on the centered versions of the vectors, $s_{i}-{\bar{s}}_{i}$ and $s_{n}-{\bar{s}}_{n}$, respectively, $CS(s_{i},s_{n})$ turns into the widely-known Pearson correlation coefficient $R(s_{i},s_{n})$:

(16) $$R\left(s_{i},s_{n}\right)=\frac{\langle s_{i}-{\bar{s}}_{i},s_{n}-{\bar{s}}_{n}\rangle }{\left|\right|s_{i}-{\bar{s}}_{i}\left||\;|\right|s_{n}-{\bar{s}}_{n}\left|\right|}=\frac{\sum_{l=1}^{L}\left(s_{l,i}-{\bar{s}}_{i}\right)\left(s_{l,n}-{\bar{s}}_{n}\right)}{\sqrt{{\sum_{l=1}^{L}(s_{l,i}-{\bar{s}}_{i})}^{2}}\sqrt{\sum_{l=1}^{L}{\left(s_{l,n}-{\bar{s}}_{n}\right)}^{2}}}$$

Unlike cosine similarity, this index is invariant to vectors shifts [24]. It finds its main application in statistics when the evaluation of mutual dependencies between two variables is needed [25,26].

Denoting with $S_{i}$ and $S_{n}$ two variables defined on an entire data population (for $S_{i}$ and $S_{n}$, vectors $s_{i}$ and $s_{n}$ represent an observed *L*-dimensional population sample), the correlation coefficient between the variables, commonly represented with the Greek letter *ρ* (instead of *R*), can be expressed by using its more general formulation:

(17) $$\rho \left(S_{i},S_{n}\right)=\frac{cov(S_{i},S_{n})}{\sigma_{S_{i}}\sigma_{S_{n}}}$$

where $cov\left(S_{i},S_{n}\right)=E\left[\left(S_{i}-\mu_{S_{i}}\right)\left(S_{n}-\mu_{S_{n}}\right)\right]$ indicates the covariance, $\mu_{S_{i}}$, $\mu_{S_{n}}$ indicate the mean and $\sigma_{S_{i}}$, $\sigma_{S_{n}}$ indicate the standard deviations of the two variables, respectively. As for cosine similarity, the correlation coefficient takes values in the interval [-1,+1], where ±1 express total positive/negative correlation, respectively, while zero means no correlation.

Correlation-based metrics have been proposed for indoor positioning [27,28]. Specifically, in the context of fingerprinting-based indoor positioning, $R(s_{i},s_{n})$ was adopted in [18] as a similarity measure for implementing a W*k*NN algorithm. More accurately, in [18], the squared value of $R(s_{i},s_{n})$, typically known as the coefficient of determination, was adopted, since positive and negative correlation are equally relevant for determining the RPs to be included in the estimation. According to this approach, the evaluated $R^{2}\left(s_{i},s_{n}\right)$ values are used for selecting the *k* RPs most relevant to the position estimation. This leads to the following position estimation formula:

(18) $${\hat{p}}_{i}=\frac{\sum_{n=1}^{k}\left(R_{i,n}^{2}\right)p_{n}}{\sum_{n=1}^{k}R_{i,n}^{2}}$$

where, for the sake of simplicity, $R_{i,n}=R\left(s_{i},s_{n}\right)$.

#### 3.2.3. A Frequentist Approach: *p*-values from the Pearson Correlation

Moving from the Pearson correlation coefficient, an additional similarity metric was proposed in [18], as part of a new method rooted in the frequentist inference theory. The method uses a well-known frequentist hypothesis test that aims at computing the degree of significance of the correlation coefficient evaluated between two sample variables [26,29]. The significance is tested under standard assumptions, and the final result is the computation of the *p*-value, which indicates the probability of observing a data sample inconsistent with the hypothesis H_0_, that the correlation $\rho (S_{i},S_{n})$ between two variables is zero, given the $R(s_{i},s_{n})$ value computed on the sample variables. A *p*-value lower than a threshold significance level $\alpha_{SL}$ indicates that H_0_ should be rejected and that the evaluated $R(s_{i},s_{n})$ has indeed a statistical significance, numerically associated with the *p*-value.

The *p*-value relative to the $R(s_{i},s_{n})$, referred to as the *p*-${\mathrm{value}}^{\left(i,n\right)}$, can be thus used as a dissimilarity metric, and RPs that are characterized by *p*-values lower than $\alpha_{SL}$ can be selected and taken into account in the following position estimation formula [18]:

(19) $${\hat{p}}_{i}=\frac{\sum_{n=1}^{k}{\left(p-{value}^{\left(i,n\right)}\right)}^{\;-1}p_{n}}{\sum_{n=1}^{k}{\left(p-{value}^{\left(i,n\right)}\right)}^{\;-1}}$$

Interestingly, it was shown in [18] that, even if the evaluation of the *p*-values leads to the same RPs selection as the Pearson correlation, the different RP weighting amplifies the gap between RPs, leading to significantly different results.

#### 3.2.4. Exploring Interdisciplinary Metrics: Shepard Similarity

Additional similarity definitions can be found by exploring different fields of knowledge, and psychology is one the most interesting fields to look at, as it focuses on the perceived similarity, defined as the degree to which two different things similarly affect people’s rational thoughts and actions, such as recognition, identification and categorization.

In this context, the multi-dimensional scaling (MDS) technique is widely used to produce a psychological space in which similarity is inversely related to distance between different stimuli [30,31,32], and the two most popular distance measures used in MDS are the Euclidean and Manhattan distances, already defined in Equation (11). In [33], Shepard proposed a simple exponential function to relate distance to similarity. Denoted with $S^{p}\left(s_{i},s_{n}\right)$, it can be generally expressed as follows:

(20) $$S^{p}\left(s_{i},s_{n}\right)=e^{-D^{p}\left(s_{i},s_{n}\right)}\;p\geq 1$$

This definition can be used for indoor positioning, for example by using Manhattan and Euclidean distances and evaluating both Shepard Manhattan-based similarity ($S^{1}\left(s_{i},s_{n}\right)$) and Shepard Euclidean-based similarity ($S^{2}\left(s_{i},s_{n}\right)$), leading to the following position estimation formula:

(21) $${\hat{p}}_{i}=\frac{\sum_{n=1}^{k}\left(S_{i,n}^{p}\right)p_{n}}{\sum_{n=1}^{k}S_{i,n}^{p}}$$

where, for the sake of simplicity, $S_{i,n}^{p}=S^{p}\left(s_{i},s_{n}\right)$.

Note that, similarly to the case of *p*-value *vs. R*, the adoption of Shepard similarity in place of Minkowski distance does not affect the RP sorting (from nearest to farthest), but it affects the RP weighting.

The use of *p*-value and Shepard similarities as RP weighting opens a further, still largely unexplored, possibility in the context of W*k*NN algorithms: a mixed approach in which the RP sorting is obtained through the use of a metric (in the presented cases, the correlation coefficient and the Minkowski distances, respectively), while different metrics, possibly obtained as a function of the previous ones, are used for the RP weighting. This possibility will be explored and investigated in Section 4.

### 3.3. A Comparative Framework for RSS-Based Similarity Metrics

The review in Section 3.2 highlighted that many different approaches exist for the definition of the similarity metric in a fingerprinting system: a natural question is then how to identify the best metric. A possibility is to measure the impact of the application of the metrics to the positioning accuracy of both flat and two-step algorithms; this approach will be followed in Section 4, with results presented and discussed in detail in Section 4.2 and Section 4.3, respectively.

In this section, however, the goal is to gain more insight into the role of the metric in identifying the most relevant RPs. Under the assumption that the goal of the positioning system is to minimize the physical distance between the actual position of a target device and its estimate, one can argue that the optimal metric is the one that sorts the RPs according to their distance from the position of the target device. This ideal metric is not obtainable in the real world, since the location of the target device is unknown by definition, but may provide a useful upper bound for the metrics defined in Section 3.2.

Given a positioning request *i*, if one defines as $p_{i}^{*}=\left(x_{i}^{*},y_{i}^{*},z_{i}^{*}\right)$ the position to be estimated and with $d(p_{i}^{*},p_{n})$ (with n=1,2,⋯,N) the spatial distances between it and each RP, such an ideal similarity metric is defined as ${\left[d\left(p_{i}^{*},p_{n}\right)\right]}^{-1}$ and leads to a sorting of RPs inversely proportional to the distance from the target position. The goodness of the metrics in Section 3.2 can be then measured by comparing the sorting of the RPs they lead to with the one resulting from the ideal metric just defined. This analysis was carried out using the testbed implemented at the DIET Department of Sapienza University of Rome, described in detail in Section 4.1.

A set of P=70 test points (TPs) and N=134 RPs was used for the analysis. For each TP, given the exact position, the spatial distances from the RPs were measured, and the ideal metric was evaluated for each RP. Next, given the TP online reading and the RP fingerprints, the RSS-based similarity metrics were computed, as well. This allowed creating, for each TP, a set of histograms, or masks, one for each similarity metric, having on the *x*-axis the RPs sorted as a function of the decreasing similarity value according to the selected metric and on the *y*-axis the spatial distance between the selected RP and the TP.

**Figure 1:**
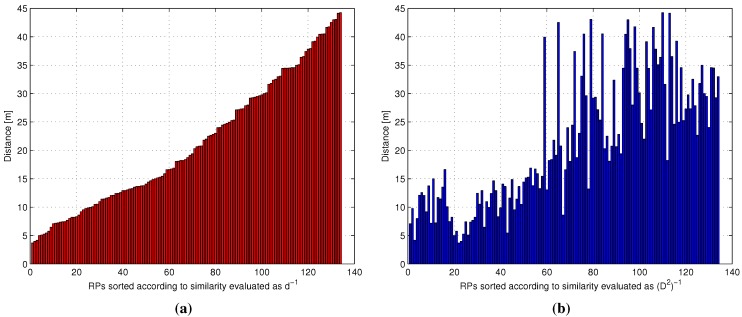
Example of reference point (RP) masks obtained for a specific test point (TP). (**a**) ${\text{M}}_{i,ideal}$, (**b**) ${\text{M}}_{i,{\left(D^{2}\right)}^{-1}}$.

As an example, Figure 1 reports the masks obtained, for a TP, for the ideal metric (Figure 1a) and for the metric defined in Equation (11) with p=2, corresponding to the Euclidean distance between the RSS vectors (Figure 1b). The histogram for the ideal metric is by definition monotonically increasing; on the other hand, the RSS-based Euclidean distance leads to a clearly different mask as a result of a different RP sorting. The difference between the masks was measured by evaluating the Euclidean distance between them element by element. By noting that, as already discussed in Section 2.2, in the application of the W*k*NN estimation formula of Equation (1) a number k<N is commonly adopted, the analysis of the masks’ difference as a function of *k* in the interval [1,N] was carried out.

Denoting with ${\text{M}}_{\;i,ideal}$ and ${\text{M}}_{i,j}$ the masks for the *i*-th TP, obtained by sorting the RPs from nearest to farthest and from most to less similar (according to the *j*-th similarity metric), respectively, the *k*-th order difference between the masks is as follows:

(22) $$\delta^{k}\left(M_{i,ideal},M_{i,j}\right)=\sqrt{\sum_{n=1}^{k}{\left(d_{ideal}\left(p_{i}^{*},p_{n}\right)-d_{j}\left(p_{i}^{*},p_{n}\right)\right)}^{2}}$$

where $d_{ideal}\left(p_{i}^{*},p_{n}\right)$ and $d_{j}\left(p_{i}^{*},p_{n}\right)$ indicate the spatial distance between the *i*-th TP and the RP occupying the *n*-th position in the masks ${\text{M}}_{i,ideal}$ and ${\text{M}}_{i,j}$, respectively. By averaging the various $\delta^{k}$ on the set of TPs, one can obtain the following indicator, expressing the *k*-th order difference between the ideal metric and the *j*-th metric:

(23) $$\Delta^{k}\left(ideal,j\right)=\frac{\sum_{i=1}^{P}\delta^{k}\left(M_{i,ideal},M_{i,j}\right)}{P}$$

**Figure 2:**
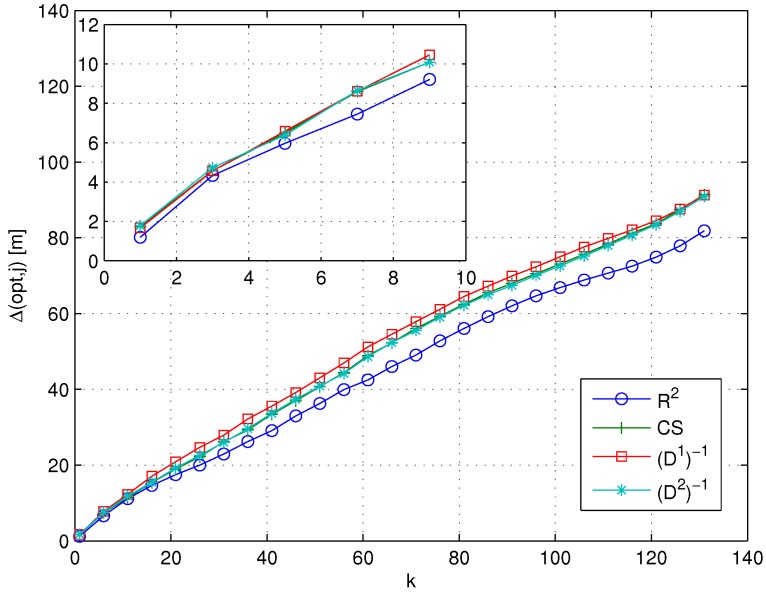
$\Delta^{k}\left(ideal,j\right)$ *vs. k* for different similarity metrics.

Figure 2 presents $\Delta^{k}\left(ideal,j\right)$ evaluated on the total set of test points, as a function of *k*, and for the similarity metrics introduced in Section 3.2 ($\Delta^{k}$ for *p*-value and Shepard similarities are not reported, since they provide, by definition, the same sorting as the correlation coefficient and the Minkowski distances, respectively, as already discussed at the end of Section 3.2). The results show that in general, the more the number of RPs (the value of *k*) taken into account in the computation of $\Delta^{k}\left(ideal,j\right)$, the higher the difference between the ideal metric and each RSS-based similarity metric. Within this general trend, different metrics lead to different performances in mimicking the sorting provided by the ideal metric. Moreover, in this particular case, the use of the coefficient of determination $R^{2}$ minimizes the set of $\Delta^{k}$, allowing one to obtain the average RPs sorting closest to the sorting provided by the use of the ideal metric. In turn, this means that the $R^{2}$ metric will lead for any given value of *k* to the selection of the set of *k* RPs that is most similar to the one selected by the ideal metric, compared to all other metrics.

Further comments on the meaningfulness of the result will be proposed in Section 4, after presenting results on positioning accuracy.

## 4. Experimental Results and Discussion

### 4.1. Testbed Implementation and Performance Indicators

**Figure 3:**
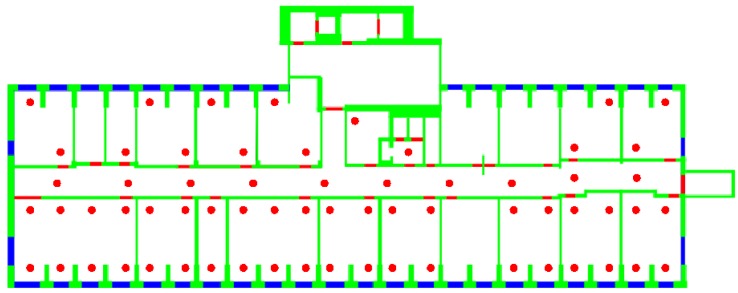
Positions of the $N_{1}$ RPs on the Department of Information Engineering, Electronics and Telecommunications (DIET) first floor.

A comparative analysis of metrics, schemes and algorithms was carried out within the experimental testbed implemented on the first two floors of the DIET Department. Each floor covers an area of 10×55 ${\text{m}}^{2}$. $N_{1}=65$ and $N_{2}=69$ RPs were identified on the first and on the second floor, respectively, for a total number of N=134 RPs, resulting in an average distance between each RP and the set of its closest RPs of approximately 3 m. Figure 3 shows, as an example, the $N_{1}$ RPs identified on the DIET first floor. In each RP, q=5 samples were taken, each sample consisting of the RSS values received from all detected APs, to counteract the impact of the channel variability on the fingerprinting database. For each AP, the values measured in the different *q* samples were then averaged. As a result, for each RP, a unique fingerprint, consisting of a list of averaged RSS values from the surrounding APs, was stored in the fingerprinting database.

Once the offline stage was completed, the total number of detected APs was L=133. No AP filtering or selection was carried out: *L* includes all WiFi signals detected in the area, including physical and virtual APs, as well as temporary and mobile connection points.

Several algorithms using both fixed and dynamic *k* selection schemes, introduced in Section 2.2, were analyzed: in particular, the fixed scheme was applied in the flat algorithm analysis (Section 4.2), studying the impact of the value of *k*, while the dynamic scheme was used in the analysis of affinity propagation-based algorithms (Section 4.3), studying the impact of the RP selection threshold at the fine localization step. In the dynamic scheme considered in the analysis, the threshold was defined by generalizing the dynamic scheme investigated in [18]:

(24) $$\lambda_{i}\left({\mathrm{sim}}_{i,n}^{F}\right)=c\cdot {\bar{\mathrm{sim}}}_{i,n}^{F}=c\cdot \frac{\sum_{n=1}^{N}{\mathrm{sim}}_{i,n}^{F}}{N}$$

where ${\bar{\mathrm{sim}}}_{i,n}^{F}$ is the average of the metric values evaluated for the *i*-th positioning request and *c* is a tuning parameter taking values in [0.1,2].

In the case of affinity propagation-based algorithms, the parameters for the matching clusters’ threshold defined in Equation (7) and used in the coarse localization phase were set to $\alpha_{1}=0.95$ and $\alpha_{2}=0.05$, in order to keep as low as possible the number of selected clusters, as suggested in [12]. Moreover, for the clustering step, 100 iterations, a DF=0.6 and a preference parameter γ=1 were adopted.

Performance was then analyzed in terms of topology, positioning accuracy and online computational complexity. Topology analysis aimed at determining the impact of different similarity metrics used for the RPs clustering steps and performance indicators, and the results are presented in Section 4.3.1.

Regarding the positioning accuracy, the positioning error $\epsilon_{i}$ was evaluated for each TP as follows:

(25) $$\epsilon_{i}=\sqrt{{\left(x_{i}^{*}-{\hat{x}}_{i}\right)}^{2}+{\left(y_{i}^{*}-{\hat{y}}_{i}\right)}^{2}+{\left(z_{i}^{*}-{\hat{z}}_{i}\right)}^{2}}$$

Moreover, assuming each $\epsilon_{i}$ as a sample of a random variable *ϵ*, the cumulative distribution function (CDF) of *ϵ* and the average error $\bar{\epsilon }=\frac{\sum_{i=1}^{P}\epsilon_{i}}{P}$ were evaluated.

Finally, regarding the computational complexity, the selected performance indicator was the number of similarity values $N_{sim}$ to be computed for obtaining the position estimate. In the case of two-step algorithms, this number for the generic *i*-th online reading can be expressed as follows:

(26) $$N_{sim}=N_{c}+N_{i}$$

where $N_{c}$ is the number of RP clusters and $N_{i}$ is the number of RPs passing the coarse localization step. Noting that in the case of flat algorithms $N_{sim}=N$ for each positioning request, one can observe that, on average, the adoption of a two-step algorithm will lead to a reduction of computational complexity if ${\bar{N}}_{sim}=N_{c}+{\bar{N}}_{i}<N$, where ${\bar{N}}_{sim}$ is the average number of similarity computations depending in turn on the average number of selected RPs ${\bar{N}}_{i}=\frac{\sum_{i=1}^{P}N_{i}}{P}$.

### 4.2. Flat Algorithms

As discussed in the comparative framework in Section 3.3, in flat algorithms, the position estimate is obtained in two steps: first, the RPs are sorted according to their similarities to the TP, and the first *k* is selected; second, the estimate is obtained as a weighted average of the positions of the *k* selected RPs, as reported in Equation (1). In order to assess the role of the similarity metrics in both RP sorting and weighting, three cases were considered:

- Ideal sorting/ideal weighting (ISIW): This case corresponds to an ideal upper bound benchmark where the spatial distances between each TP and the RPs are assumed to be known and then used for both RP sorting and weighting.
- Ideal sorting/real weighting (ISRW): In this case, the spatial distances between each TP and the RPs are assumed to be known and used during the RP sorting phase. However, once the RPs are sorted, different RSS-based metrics are evaluated as RP weights, in order to isolate the impact of RSS-based metrics on the weighting phase.
- Real sorting/real weighting (RSRW): This case represents the only feasible use case, where the spatial distances between each TP and the RPs are unknown. In this case, RSS-based metrics are evaluated and then used in both RP sorting and weighting phases.

**Figure 4:**
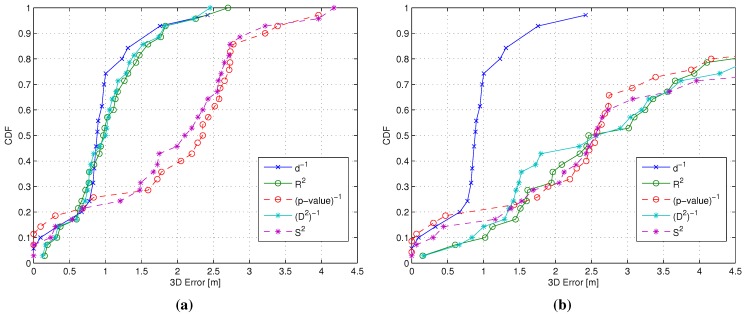
CDF of the 3D positioning error *ϵ* with k=3. ISIW, ideal sorting/ideal weighting; ISRW, ideal sorting/real weighting; RSRW, real sorting/real weighting. (**a**) ISIW ($d^{-1}$) *vs.* ISRW ($R^{2}$, (p-value${)}^{-1}$, ${\left(D^{2}\right)}^{-1}$ and $S^{2}$); (**b**) ISIW ($d^{-1}$) *vs.* RSRW ($R^{2}$, (p-value${)}^{-1}$, ${\left(D^{2}\right)}^{-1}$ and $S^{2}$).

The analysis focused on the four similarity metrics with the smallest $\Delta^{k}$ (see Section 3.3): $R^{2}$, (p-value${)}^{-1}$, ${\left(D^{2}\right)}^{-1}$ and $S^{2}$, respectively. Figure 4a shows the CDFs of *ϵ* for a W*k*NN scheme with k=3, for the ISRW case *vs.* the ISIW case. Results show that the metric used for weighting can have a significant impact on positioning accuracy, even under the ideal sorting condition, with ${\left(D^{2}\right)}^{-1}$ and $R^{2}$ metrics achieving comparable results with respect to the ISIW case, while (p-value${)}^{-1}$ and $S^{2}$ metrics cause a significant performance degradation.

**Figure 5:**
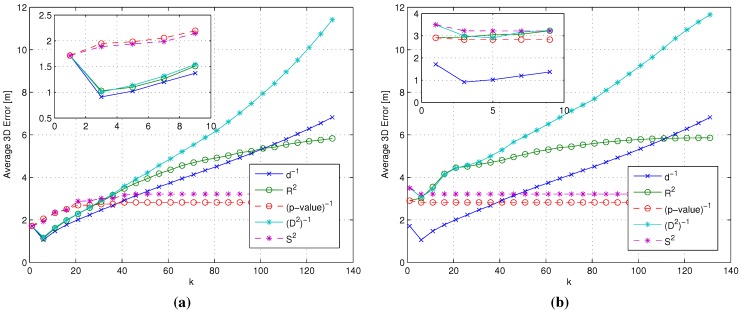
Average 3D error $\bar{\epsilon }$ as a function of *k*. (**a**) ISIW ($d^{-1}$) *vs.* ISRW ($R^{2}$, (p-value${)}^{-1}$, ${\left(D^{2}\right)}^{-1}$ and $S^{2}$); (**b**) ISIW ($d^{-1}$) *vs.* RSRW ($R^{2}$, (p-value${)}^{-1}$, ${\left(D^{2}\right)}^{-1}$ and $S^{2}$).

Figure 4b shows the CDFs of *ϵ* for a W*k*NN scheme with k=3, considering the RSRW case for the four selected RSS-based metrics again *vs.* the ISIW case, used as a benchmark. Results show that all metrics lead to significant performance degradation when compared to the ISIW case, although some metrics, in particular (p-value${)}^{-1}$ and $S^{2}$, seem to achieve slightly better performance. More insight can be obtained by analyzing the average 3D error as a function of the value of *k*, shown in Figure 5.

Figure 5a presents in fact the average 3D error for the ISRW case *vs.* the ISIW case and confirms that $R^{2}$ and ${\left(D^{2}\right)}^{-1}$ lead to an accuracy comparable to the ISIW case, at least for low values of *k*. On the other hand, Figure 5b shows that, in the RSRW case, the (p-value${)}^{-1}$ and $S^{2}$ metrics lead, for all values of *k*, to lower average errors compared to all other metrics, confirming the results in Figure 4.

Figure 4 and Figure 5 present an apparently puzzling outcome where the metrics that perform poorly in the ISRW case ((p-value${)}^{-1}$ and $S^{2}$) achieve the best results when used for both sorting and weighting. This results can be explained by observing that both (p-value${)}^{-1}$ and $S^{2}$ lead to extremely variable similarity values for different RPs, in particular when compared to the Pearson correlation and the Minkowski distance from which they are derived, respectively. This in turn means that the first most relevant RPs are characterized by similarity values much higher than the following ones, making only the first few RPs important for the position estimation. While this is a negative feature when ideal sorting is used, oppositely, it becomes a valuable feature when sorting is good, but not ideal, because the impact of sorting errors beyond the first few RPs becomes negligible thanks to the high selectivity of the weighting metric. This observation, however, only holds for metrics that guarantee a sorting as close as possible to the ideal one and confirms the relevance and the importance of the analysis carried out in Section 3.3.

### 4.3. Affinity Propagation-Based Algorithms

This section presents the experimental results for two-step algorithms based on affinity propagation. Section 4.3.1 analyzes the impact of using different similarity metrics in the RP clustering step in terms of the resulting RP topology. Section 4.3.2 moves then to focus on positioning accuracy, and finally, Section 4.3.3 analyzes the computational complexity of the online phase.

#### 4.3.1. Topology

This section compares the traditional affinity propagation metric of Equation (8), referred to in the following as gen (for generic) with the similarity metrics introduced in Section 3.1 and Section 3.2, by studying how they affect the definition of clusters, their number and their size.

**Figure 6:**
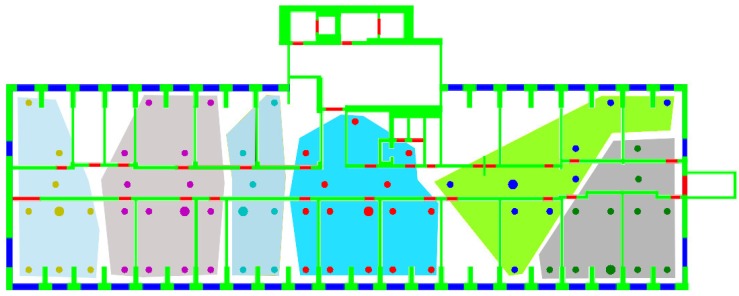
RP clusters and exemplars obtained on the DIET first floor with gen as the similarity metric (areas represented in different colors indicate different clusters; larger dots indicate exemplars).

Before moving to the discussion of the results, it is worth pointing out the following details about the implementation of the affinity propagation algorithm:

- Clustering was performed separately for the two floors composing the testbed, assuming the knowledge of the floor for each RP.
- With reference to the degeneracy issue identified in Section 2.3.1, the solution proposed in [13] of adding random noise to similarity values was not implemented, since different similarities are characterized by significantly different ranges of values, making it impossible to add noise with the same power to all similarity metrics.
- On the other hand, the outlier issue, also identified in Section 2.3.1, was addressed by actually implementing the solution proposed in [12] to eliminate such outliers. This leads in all cases to clusters occupying convex regions on each floor. Figure 6 shows as an example the clusters obtained on the DIET first floor, using the gen metric.

The following indicators where considered in the comparison: the number of formed clusters $N_{c}$, the average number of RPs within a cluster ($\bar{\left|C\right|}$), the maximum and minimum cluster cardinalities (max{|C|} and min{|C|}) and the variance of the cluster cardinality (var{|C|}). Results for all metrics are presented in Table 1.

**Table 1:** RP clustering via affinity propagation.

Metric	$N_{c}$	$\bar{\left|C\right|}$	max{|C|}	min{|C|}	var{|C|}
$d^{-1}$	37	3.62	6	2	1.07
gen	13	10.31	15	5	9.06
$R^{2}$	13	10.31	19	4	27.73
(p-value${)}^{-1}$	43	3.11	6	2	1.10
CS	13	10.31	15	5	7.90
${\left(D^{1}\right)}^{-1}$	29	4.62	8	2	3.17
${\left(D^{2}\right)}^{-1}$	27	4.96	8	2	4.19
$S^{1}$	34	3.94	7	2	2.54
$S^{2}$	20	67	69	2	216.01

Results show that metrics can be approximately divided into two groups:

- The first group including gen, $R^{2}$ and CS metrics, leading to the creation of relatively few, large clusters, although with different variances.
- The second group, including $d^{-1}$, (p-value${)}^{-1}$, ${\left(D^{1}\right)}^{-1}$, ${\left(D^{2}\right)}^{-1}$ and $S^{1}$ metrics, leading to small clusters with low variances. (p-value${)}^{-1}$ in particular leads to the largest number of clusters with the lowest variance among all metrics.

It is worth pointing out that results for $S^{2}$ highlight that this similarity metric did not converge on the second floor after one hundred iterations, showing an example of the impact of degeneracies in the application of different similarity metrics to clustering based on affinity propagation.

Results shown in Table 1 raise the natural question of what is the best metric for RP clustering. In order to answer this question, one could resort to adopting a clustering goodness indicator, such as net similarity, widely used for clustering scheme comparisons [13]. When applied to Table 1, net similarity would indicate that the first group of metrics (gen, $R^{2}$ and CS) should be the preferred choice for clustering. It is, however, worth observing that a high net similarity value does not necessarily correspond to a good performance in terms of positioning accuracy, as will be further discussed in Section 4.3.2.

#### 4.3.2. Positioning Accuracy: A Backward Approach

This section focuses on the positioning accuracy achievable by combining different metrics for clustering, coarse and fine localization steps, respectively.

In the following, for the ease of notation, a generic combination will be indicated with the triplet $m_{1}/m_{2}/m_{3}$ where $m_{1}$ indicates the similarity metric used in the clustering step and previously referred to as ${\mathrm{sim}}^{CL}\left(.,.\right)$, $m_{2}$ indicates the coarse localization metric, previously referred to as ${\mathrm{sim}}^{C}\left(.,.\right)$, and, finally, $m_{3}$ indicates the fine localization metric, previously referred to as ${\mathrm{sim}}^{F}\left(.,.\right)$.

For the sake of the clarity of exposition, not all of the different combinations obtainable by selecting different metrics for the three different steps will be presented in the following. Rather, the following backward approach was adopted:

1. Cluster matching criterion selection: The first step in the backward approach was the selection of one of the cluster matching criteria introduced in Section 2.3.2 in order to focus on a single criterion in the following steps of the analysis. In order to do so, both $m_{1}$ and $m_{2}$ were set equal to the gen metric, while the four metrics characterized by the smallest $\Delta^{k}$, already considered in the analysis of flat algorithms (see Section 3.3 and Section 4.2), were adopted as the $m_{3}$ metric, in order to identify which of the two criteria performs better under different conditions.
2. $m_{2}/m_{3}$ selection: Having selected the best cluster matching criterion, the second step focused on the analysis and possibly the selection of the best metrics to be used during both coarse and fine localization steps. To do so, the $m_{1}$ metric was kept set to the gen metric, while $m_{2}$ and $m_{3}$ metrics were allowed to change. In particular, all of the RSS-based metrics introduced in Section 3.2 were considered as candidates for the role of the $m_{2}$ metric, while, based on the analysis already carried out in Section 3.3 and Section 4.2, candidates for the role of the $m_{3}$ metric were restricted to the four metrics with the smallest $\Delta^{k}$. Based on the positioning errors achieved by the different gen/$m_{2}/m_{3}$ combinations, this phase concluded with a joint $m_{2}$ and $m_{3}$ selection.
3. $m_{1}$ selection: The last step was the analysis of the impact of different metrics on the RP clustering step, aiming at the selection of the best one. In this case, all metrics defined in both Section 3.1 and Section 3.2 were eligible for the role of the $m_{1}$ metric, while keeping both $m_{2}$ and $m_{3}$ set to the metrics selected as a result of the $m_{2}/m_{3}$ selection step.

Throughout the section, the performance of the flat algorithm using the same similarity metrics adopted as $m_{3}$ in the analysis of two-step algorithms will be presented as a reference benchmark. 

*Step 1: Cluster* *Matching Criterion Selection*

**Figure 7:**
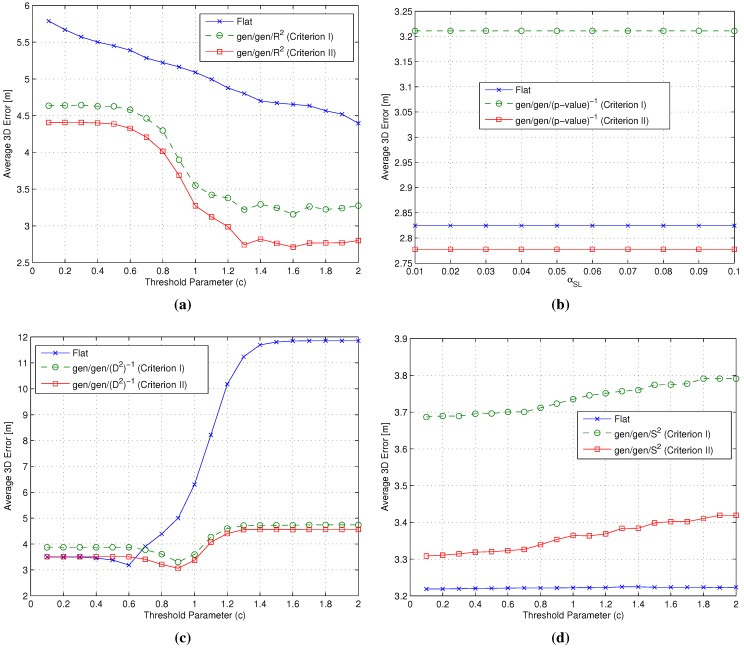
Impact of the matching cluster Criteria I/II on the average 3D error $\bar{\epsilon }$. (**a**) flat *vs.* gen/gen/$R^{2}$ combinations; (**b**) flat *vs.* gen/gen/(p-value${)}^{-1}$ combinations; (**c**) flat *vs.* gen/gen/${\left(D^{2}\right)}^{-1}$ combinations; (**d**) flat *vs.* gen/gen/$S^{2}$ combinations.

Figure 7 presents the average error $\bar{\epsilon }$, obtained by varying the threshold parameter *c* (or the significance level $\alpha_{SL}$, in the case of the *p*-value algorithm [18]), for the gen/gen/$m_{3}$ metrics’ combinations and the flat algorithm adopting the same $m_{3}$ metric. Precision as measured by the standard error was in the worst case equal to ±0.05 m.

Results show that Criterion II performs slightly better than Criterion I for each gen/gen/$m_{3}$ combination, suggesting that the adoption of an average cluster RSS fingerprint as representative of the cluster is the best option in order to maximize the positioning accuracy. As a result, Criterion II was selected and used in the following steps of the backward analysis. As an interesting side note, results also show that two-step algorithms achieved comparable or better performance than the flat ones for all considered metrics.

*Step 2:* $m_{2}/m_{3}$ *Selection*

**Figure 8:**
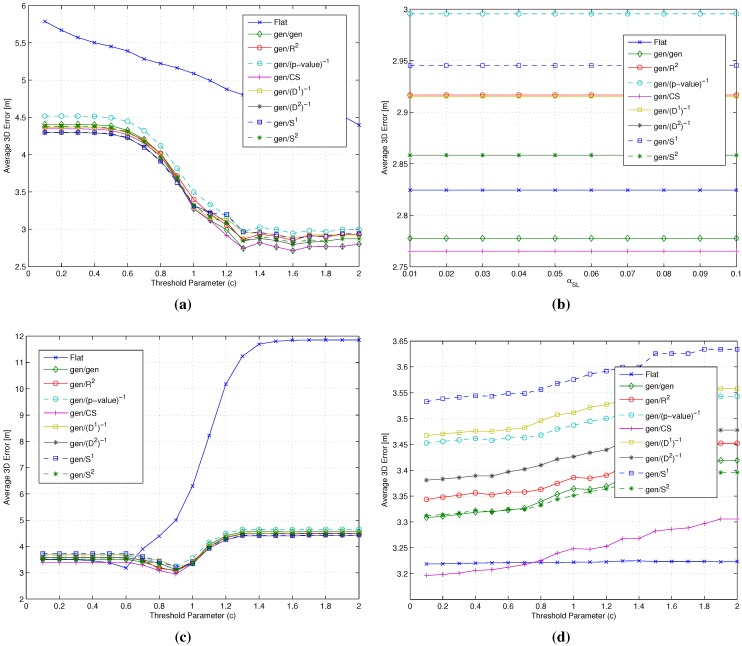
Impact of the $m_{2}$ and $m_{3}$ metrics on the average 3D error $\bar{\epsilon }$. (**a**) flat *vs.* gen/$m_{2}$/$R^{2}$ combinations; (**b**) flat *vs.* gen/$m_{2}$/(p-value${)}^{-1}$ combinations; (**c**) flat *vs.* gen/$m_{2}$/${\left(D^{2}\right)}^{-1}$ combinations; (**d**) flat *vs.* gen/$m_{2}$/$S^{2}$ combinations.

Figure 8 shows the average error $\bar{\epsilon }$, for the flat algorithm *vs.* the gen/$m_{2}$/$m_{3}$ metrics’ combinations. Precision as measured by the standard error was in the worst case equal to ±0.04 m.

Firstly, one can observe that results again show that two-step algorithms always lead to comparable or better results with respect to the flat ones. The performance increase provided by the two-step algorithms is in particular significant when the selected value of *c* leads to a low selection threshold and, thus, to a large number of RPs being selected. Under these conditions, the RP space reduction provided by clustering leads to significant reduction in the value of $\bar{\epsilon }$.

Moving to the analysis of the impact of the different metrics on the performance of the two-step algorithms, results show that the (p-value${)}^{-1}$ and $S^{2}$ metrics lead to a substantial independence of the performance from the value of the selection threshold, as shown in Figure 8b,d. This is due to the selective feature of these metrics, already discussed in Section 4.2, which leads to a small number of RPs being relevant in the position estimation.

**Figure 9:**
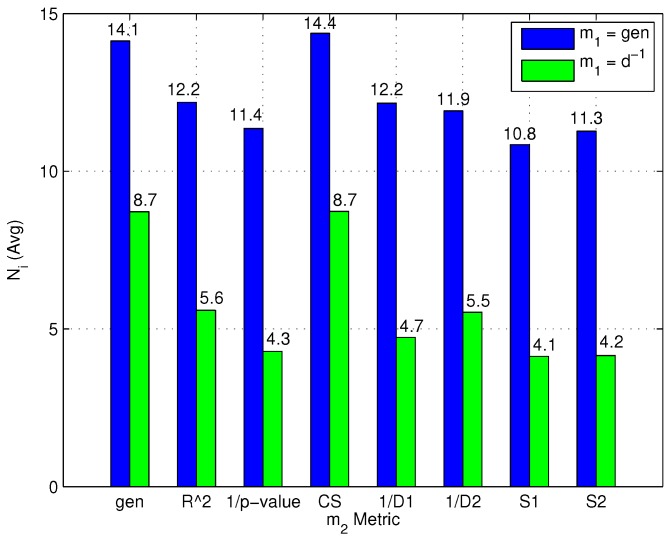
Impact of $m_{1}$ and $m_{2}$ metrics on ${\bar{N}}_{i}$.

The results shown in Figure 8 lead furthermore to the following conclusions:

- Impact of the $m_{3}$ metric: The use of different $m_{3}$ metrics significantly affects the positioning accuracy. Among all metrics, (p-value${)}^{-1}$ emerged as the best candidate to play the role of $m_{3}$, since at the same time, it minimizes the impact of the RP selection threshold and the positioning error, with a value around 2.76 m below any other metric.
- Impact of the $m_{2}$ metric: Different $m_{2}$ metrics have a negligible effect on the positioning accuracy, with a difference in the evaluated average errors in a range of about twenty centimeters. The substantial independence of the performance from the selected $m_{2}$ metric is confirmed by Figure 9, showing the impact of metrics on the value of ${\bar{N}}_{i}$ (defined in Section 4.1 as the average number of RPs within the clusters selected after the coarse localization step).

Figure 9 focuses in fact on the impact of $m_{1}$ and $m_{2}$ on ${\bar{N}}_{i}$ and shows that once the $m_{1}$ metric is selected, the choice of $m_{2}$ has a limited impact on ${\bar{N}}_{i}$.

As a results of this step of the backward analysis, the (p-value${)}^{-1}$ metric was selected as the $m_{3}$ metric, while the gen metric was kept as $m_{2}$, in accordance with previous literature, given the fundamental independence of the performance from this metric.

*Step 3:* $m_{1}$ *Selection*

**Figure 10:**
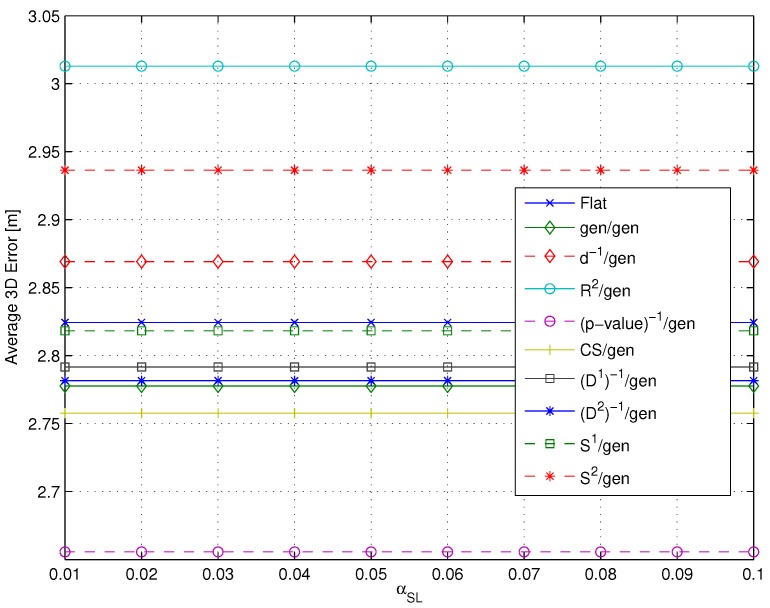
Impact of the $m_{1}$ metric on the average 3D error $\bar{\epsilon }$: flat *vs.* $m_{1}$/gen/(p-value${)}^{-1}$ combinations.

Figure 10 shows the average error $\bar{\epsilon }$ for the flat algorithm *vs.* the $m_{1}$/gen/(p-value${)}^{-1}$ combinations. Precision as measured by the standard error was in the worst case equal to ±0.03 m.

**Figure 11:**
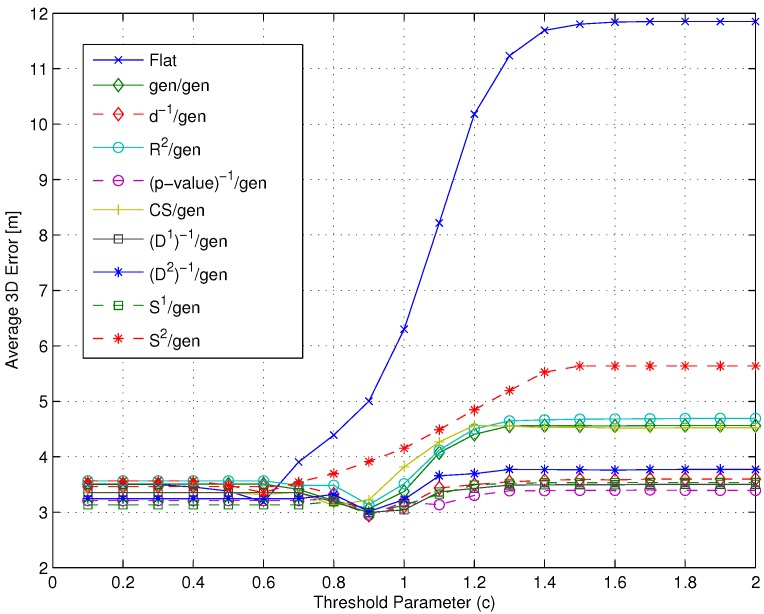
Impact of the $m_{1}$ metric on the average 3D error $\bar{\epsilon }$: flat *vs.* $m_{1}$/gen/${\left(D^{2}\right)}^{-1}$ combinations.

Figure 10 shows that among all possible combinations, the (p-value${)}^{-1}$/gen/(p-value${)}^{-1}$ one leads to the best positioning accuracy with an average 3D error of about 2.65 m. All metrics lead, however, to positioning errors in the order of 3 m. As a consequence of the results in Figure 10, the conclusion was to select (p-value${)}^{-1}$ as the $m_{1}$ metric. This result is also confirmed by an exhaustive search over all possible $m_{1}/m_{2}/m_{3}$ combinations, not shown here graphically for the sake of clarity. In order to confirm this conclusion, however, in Figure 11, results obtained for different $m_{1}$ metrics with $m_{2}=$ gen and $m_{3}=$${\left(D^{2}\right)}^{-1}$ are presented (worst case precision: ±0.03 m) and confirm that a different $m_{3}$ metric leads to worse positioning accuracy than the (p-value${)}^{-1}$, for all considered $m_{1}$ metrics. Two additional observations can be derived from the results presented in this section:

- Figure 11 shows that if a $m_{3}$ metric different from (p-value${)}^{-1}$, characterized by a lower selectivity, is selected, the impact of $m_{1}$ can be significantly larger, especially when many RPs are selected.
- Both Figure 10 and Figure 11 highlight that the adoption of the only spatial distance-based metric $d^{-1}$ as the $m_{1}$ metric has no particular impact on performance.

#### 4.3.3. Computational Complexity

It was previously stated in the paper that one of the main expected advantages of two-step algorithms is the reduction of the computational complexity of the online phase. In order to assess this aspect, the value of ${\bar{N}}_{sim}$ defined in Section 4.1 was measured for both flat and two-step algorithms as a function of the $m_{1}$ metric. It is worth noting that the $m_{1}$ metric is the only relevant metric in this analysis, since $m_{2}$ and $m_{3}$ have no impact on the number of clusters.

**Figure 12:**
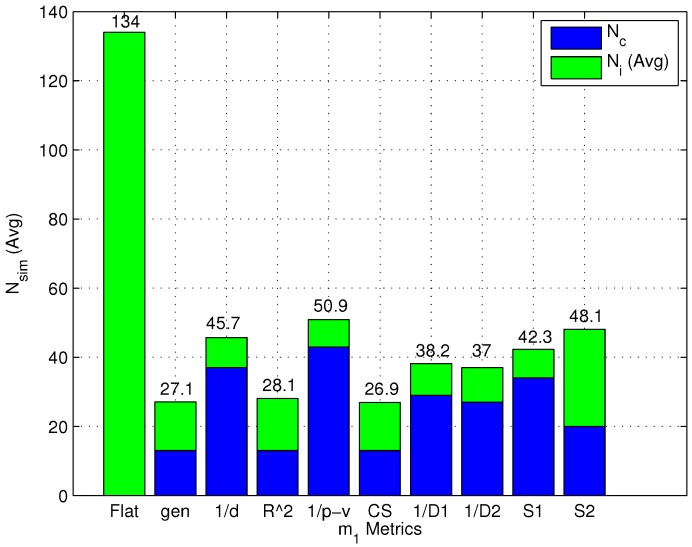
${\bar{N}}_{sim}$ for both flat and two-step algorithms (with several $m_{1}$ metrics).

Figure 12 shows the values of ${\bar{N}}_{sim}$ for both flat and two-step algorithms as a function of the $m_{1}$ metric. For two-step algorithms, Figure 12 also highlights the contributions of $N_{c}$ and ${\bar{N}}_{i}$ to ${\bar{N}}_{sim}$, for each $m_{1}$ metric, respectively.

The following observations can be made based on the results in Figure 12:

- As expected, the number of computed similarity values is always equal to *N* for a flat algorithm.
- Two-step algorithms significantly reduce the average computational complexity of the online phase. Moreover, $m_{1}$ metrics can be divided into two different groups: (1) $m_{1}$ metrics that minimize the number of formed clusters and conversely maximize the number of RPs selected after the coarse localization step; and (2) $m_{1}$ metrics that maximize the number of formed clusters and conversely minimize the number of RPs remaining after the coarse localization step.
- The first group of metrics shows a ${\bar{N}}_{sim}$ value slightly lower than the second group. CS in particular obtained the lowest computational complexity; at the same time, both groups show a ${\bar{N}}_{sim}$ value significantly lower than the flat algorithm.
- As a final note, it can be observed that the $m_{1}=(p$-value${)}^{-1}$ metric, which minimizes positioning error as found in Section 4.3.2, is not the metric minimizing ${\bar{N}}_{sim}$.

## 5. Conclusions

In this work, a comprehensive review of W*k*NN WiFi fingerprinting-based indoor positioning algorithms has been provided. The role of the similarity metric between the RP fingerprints and online readings was investigated, by considering both flat W*k*NN algorithms and two-step algorithms based on affinity propagation.

For two-step algorithms, the impact of the similarity metric in three different steps involved in determining the position estimate, that is clustering, coarse localization and fine localization, was analyzed, and a novel mixed approach combining different metrics at different steps was proposed and investigated.

The extensive experimental analysis carried out in this work highlighted that two-step algorithms provided an effective solutions for improving the performance of flat algorithms in terms of both positioning accuracy and computational complexity. The analysis also highlighted that combining different metrics for the different steps of the two-step algorithm is a viable and promising solution to improve the positioning accuracy. In particular, the best results were obtained by metrics that lead to a large number of small clusters of RPs in the clustering step and at the same time are highly selective in the fine localization step. Finally, a trade-off was identified between positioning accuracy and computational complexity in which a slight performance decrease must be accepted for one of the two performance indicators in order to optimize the system with respect to the other.

Future works will focus on extending the presented analysis by considering different environments, other clustering algorithms and an extended set of deterministic similarity metrics. A second interesting research direction is the extension of the work to the case of probabilistic similarity metrics. A third research line that was identified is related to the removal of the assumption of the knowledge of the floor of each RP during the clustering step, allowing the formation of 3D clusters. In parallel, a study of the reduction of complexity in the offline phase through the introduction of virtual RP fingerprints estimated by means of indoor channel modeling was recently initiated [34].
